# Support Values for Genome Phylogenies

**DOI:** 10.3390/life6010011

**Published:** 2016-03-07

**Authors:** Fabian Klötzl, Bernhard Haubold

**Affiliations:** Department of Evolutionary Genetics, Max-Planck-Institute for Evolutionary Biology, August-Thienemann-Straße 2, 24306 Plön, Germany; kloetzl@evolbio.mpg.de

**Keywords:** bootstrap, quartet analysis, support value, phylogeny, distance matrix

## Abstract

We have recently developed a distance metric for efficiently estimating the number of substitutions per site between unaligned genome sequences. These substitution rates are called “anchor distances” and can be used for phylogeny reconstruction. Most phylogenies come with bootstrap support values, which are computed by resampling with replacement columns of homologous residues from the original alignment. Unfortunately, this method cannot be applied to anchor distances, as they are based on approximate pairwise local alignments rather than the full multiple sequence alignment necessary for the classical bootstrap. We explore two alternatives: pairwise bootstrap and quartet analysis, which we compare to classical bootstrap. With simulated sequences and 53 human primate mitochondrial genomes, pairwise bootstrap gives better results than quartet analysis. However, when applied to 29 *E. coli* genomes, quartet analysis comes closer to the classical bootstrap.

## 1. Introduction

Early phylogenies came without significance tests. It thus remained unclear whether the reconstructed tree was significantly better than an alternative tree or how reliably individual nodes would be recovered if a new set of characters were sampled. Of these two types of analyses, assessing whole trees *vs.* assessing individual clades of a given tree, it is the latter that is most commonly carried out. And among the methods available for doing this, the bootstrap is the most widely used [1].

The bootstrap is a simple, but highly effective method for solving the following problem in statistics: given a sample of *n* measurements, what is the distribution of, say, the mean of these measurements if we do not know the null distribution from which the original measurements were drawn. The solution using the bootstrap consists of drawing *n* measurements with replacement from the original sample and recalculating the statistic of interest; the mean in our example [2]. By repeating this many times, the null distribution of the statistic is generated, which can be compared to another sample in order to test the null hypothesis that the two samples were drawn from the same population [3].

This example shows two things: first, the bootstrap is only practical if computing is inexpensive, as it has been since the introduction of the PC in the mid-1980s. Second, in the limit of a large sample size, bootstrap samples become identical to the original sample.

**Figure 1:**
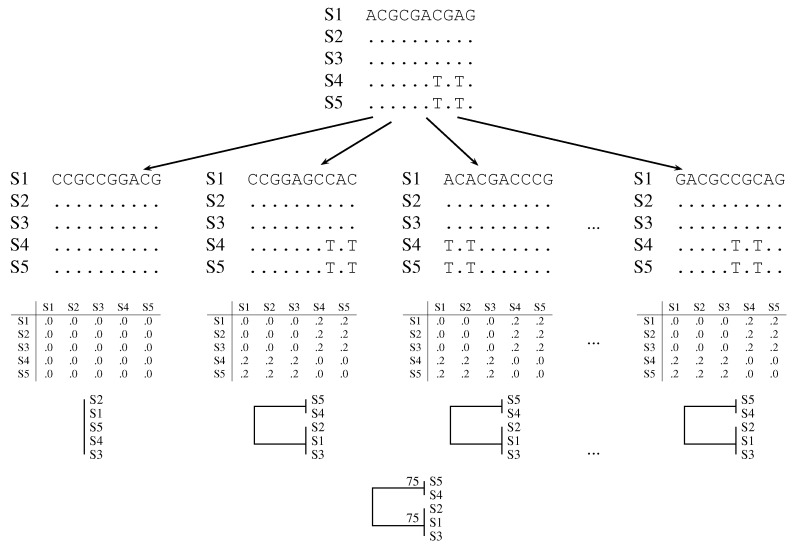
Cartoon of classical bootstrap. The columns of the original alignment (top row) are repeatedly resampled with replacement (second row). Distance matrices are computed from the bootstrap samples (third row) and summarized as phylogenies (fourth row). The clades in the bootstrapped phylogenies are summarized in a consensus tree with support values written next to the nodes (fifth row). A dot indicates a match to the nucleotide in the top row.

Felsenstein introduced the bootstrap in phylogeny reconstruction [4]: Consider an alignment of DNA sequences as an *m* by *n* matrix of nucleotides, where rows represent taxa and columns represent homologous residues (Figure 1, top row). Compute a tree from this data matrix. Then, construct a pseudo-sample by drawing with replacement *n* columns from the original sample. This pseudo-sample is called a bootstrap sample. Compute the tree from the bootstrap sample and repeat this many times. Record the number of times each clade of the original tree appears in the bootstrapped trees. This value is called the bootstrap support value (Figure 1, bottom row).

Assigning bootstrap values to individual nodes has become standard practice in alignment-based phylogeny reconstruction. However, computing alignments of very long sequences, such as the megabase-sized genomes of bacteria or the gigabase-sized genomes of mammals, is computationally demanding. Nevertheless, an increasing number of bacterial outbreaks are being tracked by whole genome sequencing. For example, 3085 strains of *Streptococcus pneumoniae*, each 2.2 Mb long, were sequenced during an outbreak of this human pathogen [5]. A quick way to cluster sequence samples of this magnitude is highly desirable.

Perhaps surprisingly, such clustering can be carried out without alignment [6,7]. Now, without alignment, the original bootstrap can no longer be applied as it relies on resampling columns of homologous nucleotides. However, one might argue that for megabase-long sequences and beyond, the bootstrap reaches the limit in which it cannot generate any useful variation.

Here, we investigate this problem for our recently-published distance estimation program andi [8]. It computes distances from approximate pairwise local alignments. Using suffix arrays, these approximate pairwise alignments can be computed very quickly; for example, 3085 *S. pneumoniae* strains are clustered on an 24-core computer in 4:37 h using 9.2 GB of RAM. However, the classical bootstrap is not applicable to pairwise alignments, and we propose two alternatives: pairwise bootstrap and quartet analysis. Pairwise bootstrap is a new variant of the Felsenstein bootstrap, while quartet analysis, which evaluates the agreement between a phylogeny and the underlying distance matrix, is taken from the literature [9]. We explore both methods by comparing them to the classical bootstrap when applied to simulated datasets, where pairwise bootstrap clearly outperforms quartet analysis. We also analyze two empirical datasets. The first comprises 53 human mitochondrial genomes, which are relatively short with only 16.6 kb each. The second dataset contains 29 complete *E. coli*/*Shigella* genomes, which are roughly 300-times longer than the mitochondrial genomes. Pairwise bootstrap outperforms quartet analysis when applied to the mitochondrial genomes. However, the converse is true for the *E. coli* dataset.

## 2. Methods and Data

### 2.1. Classical Bootstrap

An alignment consists of *m* rows of nucleotides, corresponding to taxa, and *n* columns, corresponding to homologous residues. Given such an alignment, we compute the “classical” bootstrap by resampling columns with replacement and recomputing a matrix of Jukes–Cantor distances. This procedure is implemented in our program dnaDist, the sources and documentation of which are available from the website accompanying this paper:
http://evolbioinf.github.io/life2015

### 2.2. Quartet Analysis

**Figure 2:**
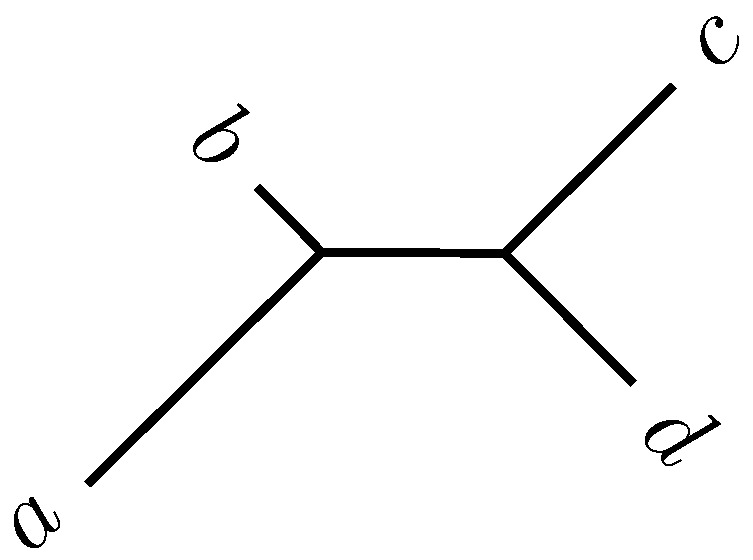
Phylogeny for four taxa.

Consider four taxa, a,b,c,d, connected by an unrooted phylogeny (Figure 2). Their pairwise distances are given as $d_{x,y}$. The topology shown in Figure 2 is correct if *a* is most closely related to *b* and *c* to *d*. This condition is known more formally as the four-point criterion:

$$d_{a,b}+d_{c,d}\leq \min\left(d_{a,c}+d_{b,d},d_{a,d}+d_{b,c}\right)$$

A set of four taxa fulfilling the four-point criterion is a supporting quartet. The support value for an edge is the proportion of supporting quartets that traverse it. The published program PhyD* implements quartet analysis [10].

Quartet analysis is time consuming, because the traversal of all quartets takes time $O(n^{4})$ for each of *n* internal edges. Hence, quartet analysis is expected to run in time $O(n^{5})$. This prompted us to re-implement quartet analysis in our program afra with a view toward maximizing efficiency.

### 2.3. Pairwise Bootstrap

**Figure 3:**
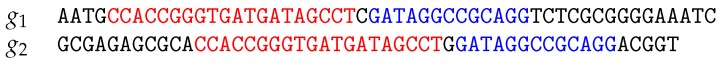
Illustration of anchors marked in red and blue for computing the anchor distance between the toy genomes $g_{1}$ and $g_{2}$.

Anchor distances are based on long exact matches between pairs of genomes that flank regions containing mismatches. An anchor is a unique exact match between two genomes of length ≥l, where *l* is the smallest value that makes it unlikely to find a match of this length by chance alone [8]. Figure 3 shows an example pair of genomes, $g_{1}$ and $g_{2}$. Genome $g_{1}$ contains two anchors with the matches in $g_{2}$ displayed in corresponding colors. The anchors have the same distance in $g_{1}$ and $g_{2}$, a single nucleotide, and are thus regarded as an approximate local alignment. The mismatches per site between $g_{1}$ and $g_{2}$ are then computed as one divided by the number of nucleotides covered by the red and blue anchors plus the intervening nucleotide, that is 1/(20+13+1)=0.029. This is Jukes–Cantor corrected to yield the anchor distance of 0.031. The computation of anchor distances is implemented in our program andi [8].

**Figure 4:**
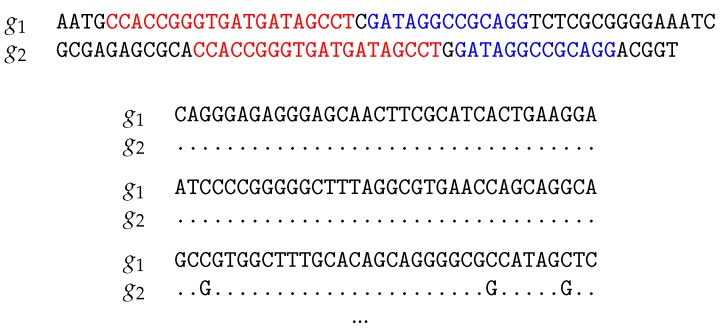
Pairwise bootstrap samples based on the anchors shown in Figure 3. Dots indicate matching nucleotides.

We bootstrap the local alignments implied by the anchor distances and call this *pairwise bootstrap* (Figure 4). To implement pairwise bootstrap, let $m_{ij}$ be the number of mismatches found among all approximate local alignments between sequences *i* and *j*, and let $n_{ij}$ be the number of nucleotides covered by these alignments. Then, the number of mismatches per site, $p_{ij}=m_{ij}/n_{ij}$, is the probability of drawing a mismatch from the $n_{ij}$ nucleotides. Let $m_{ij}^{\prime }$ be the number of mismatches found after sampling with replacement $n_{ij}$ times the $n_{ij}$ homologous sites. $m_{ij}^{\prime }$ is a random variable drawn from a binomial distribution parameterized by $n_{ij}$ trials and success probability $p_{ij}$. Therefore, instead of actually carrying out the bootstrap as illustrated in Figure 4, which becomes slow for long sequences, we instantaneously draw $m_{ij}^{\prime }$ from a binomial distribution. For each $p_{ij}$, we compute its bootstrapped version as $p_{ij}^{\prime }=m_{ij}^{\prime }/n_{ij}$ to generate pseudo-sampled distance matrices. Any position-independent distance metric, e.g., Jukes–Cantor or Kimura two-parameter [11], can be calculated in this way.

### 2.4. Simulation

The agreement between classical bootstrap, on the one hand, and pairwise bootstrap and quartet analysis, on the other, was assessed using the following simulation scheme. All programs not referenced are written by us and can be reached via the website accompanying this paper.

1. Simulate *n* related sequences using the coalescent simulator ms [12].
2. Convert the output of ms to an alignment of DNA sequences, *A*, using ms2dna.
3. Subject *A* to bootstrap analysis using dnaDist.
4. Compute the consensus tree and support values from the output of dnaDist using the program consense, which is part of the PHYLIP package [13].
5. Subject *A* to pairwise bootstrap analysis as implemented in the latest version of andi [8] and also calculate the consensus tree using consense.
6. Use afra to carry out quartet analysis on andi-distances computed from *A*.
7. For each cluster in the consensus tree, extract the three support values classical, pairwise and quartet using the program correlation.js.
8. Repeat.

The panels in Figure 7 were generated from the output of this simulation by computing for each classical bootstrap value the average value of the two alternative support values.

### 2.5. Resource Consumption

All computations were carried out on a system with 24 Intel Xeon cores running at 2.60 GHz under the Linux distribution Ubuntu 14.04 LTS. Time and memory consumption was measured using commands like

/usr/bin/time -f “elapsed\t%Es\nuser\t%Us\nmem\t%MkB\n” \
andi -b 1000 foo.fasta > foo.dist

for pairwise bootstrap analysis, and

/usr/bin/time -f “elapsed\t%Es\nuser\t%Us\nmem\t%MkB\n“ \
java -Xmx4096m -jar PhyDstar.jar -c -i foo.dist

for PhyD* [10].

### 2.6. Data

Apart from simulated data, we compared the performance of bootstrapped anchor distances and alignment-based bootstrap distances by analyzing two example datasets: The first consisted of 53 human mitochondrial genomes [14] with an average length of 16.6 kb. The second dataset consisted of 29 genomes of *Escherichia coli* and *Shigella* with an average length of 4.9 Mb. Both datasets are also posted on the project website.

### 2.7. Alignment and Phylogeny Computation

The 53 human mitochondrial genomes were aligned using clustalw [15]. The 29 *E. coli*/*Shigella* genomes were aligned using the fast genome aligner mugsy [16]. As described for the simulations (Section 2.4), Jukes–Cantor distances were computed and bootstrapped using dnaDist, and the distance matrices were subjected to neighbor joining as implemented in the program clustDist. Consensus trees were computed using the PHYLIP program consense [13], and the output of two consense runs was compared using the program correlation.js. Trees were midpoint-rooted using retree, which is also part of PHYLIP.

## 3. Results and Discussion

### 3.1. Resource Consumption

#### Pairwise Bootstrap

**Figure 5:**
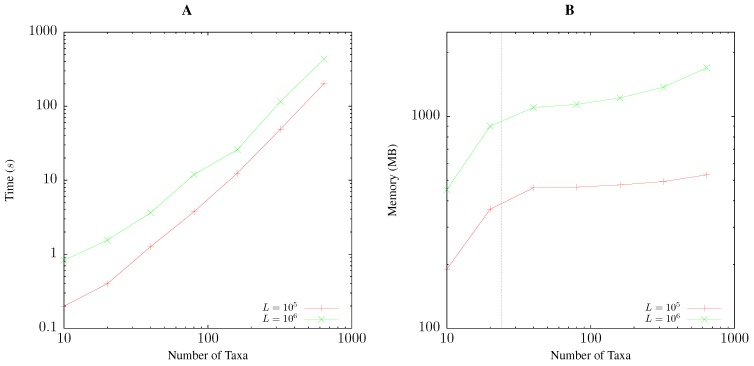
Time (**A**) and memory (**B**) required by andi to compute 1000 bootstrap replicates as a function of the number of taxa for sequences of 100 kb and 1 Mb length, *L*. The vertical line in (**B**) is at 24, the number of cores on the test computer.

Pairwise bootstrap takes time proportional to the size of the distance matrix. Accordingly, Figure 5A shows for each doubling of the number of taxa a quadrupling of the run time. On the test computer, 100 taxa with 1-Mb sequences took approximately 15 s. The memory consumption of pairwise bootstrapping is dominated by the parallelized suffix array computation underlying the calculation of anchor distances [8]. Hence, the memory requirement grows linearly up to 24 taxa, the number of cores on the test machine, and then levels off, only to pick up again when storing the raw sequence data results in appreciable memory consumption as seen for 640 taxa each with 1 Mb (Figure 5B). This requires approximately 1.7 GB of memory. 

#### Quartet Analysis

**Figure 6:**
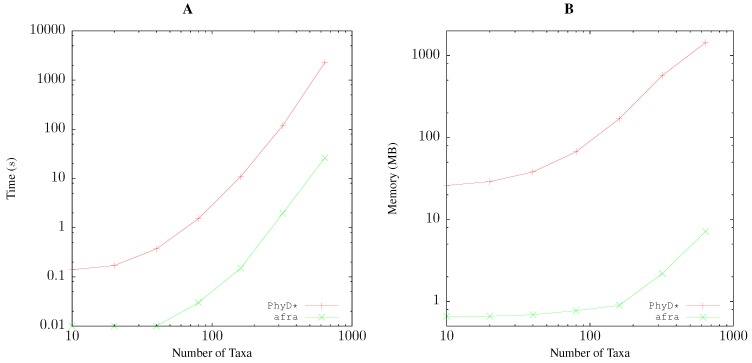
Comparing time (**A**) and memory (**B**) consumption between two implementations of quartet analysis, the published PhyD* [10] and our own program, afra, when applied to distance matrices of varying size.

The run time of quartet analysis *given* a distance matrix also grows polynomially with the number of taxa. Figure 6A shows that our program afra takes approximately 0.04 s for 100 taxa, while the reference implementation, PhyD* [10], requires approximately 4 s, that is 100-times longer. However, both programs roughly increase their run time ten-fold for a doubling of the number of taxa. This deviates substantially from the theoretical $O(n^{5})$ run time, according to which a doubling of sample size should result in a 32-fold increase in run time. We do not know the reason for this discrepancy, but it illustrates the importance of empirical resource measurements when analyzing software.

The memory consumption of afra is less than 1 MB for 100 taxa, while PhyD* uses approximately 100 MB for the same number of taxa (Figure 6B). However, memory consumption grows with similar rates for both applications.

To test the scalability of quartet analysis, we applied it to a sample of 3085 genomes of *Streptococcus pneumoniae*, which were sequenced in the course of a pneumococcal outbreak [5]. We calculated the corresponding pairwise distance matrix and neighbor-joining tree using andi in 4:37 h. Quartet analysis then took 2:18 h and occupied 150 MB of memory. This shows that quartet analysis scales well to large datasets.

### 3.2. Accuracy

**Figure 7:**
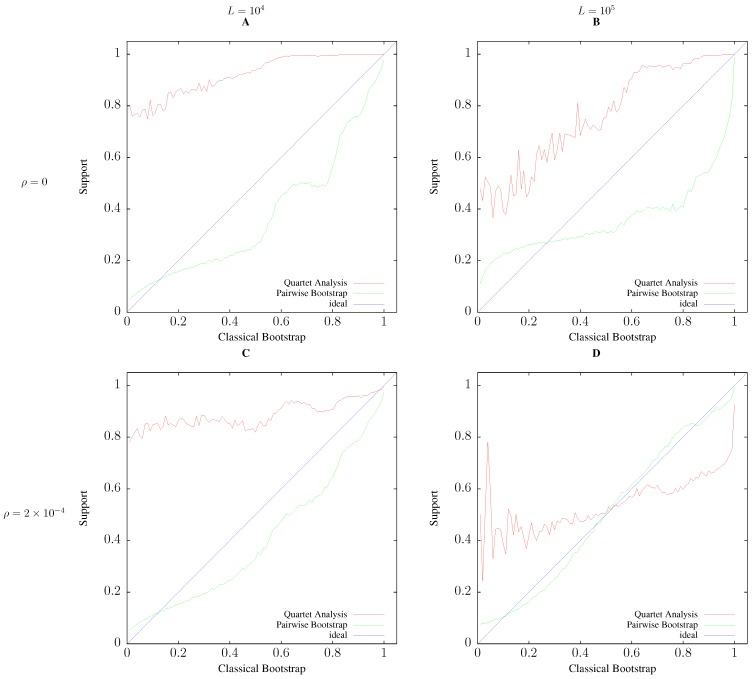
Average support values as a function of classical bootstrap support. All simulations with sample size n=20, 1% polymorphisms per position, and $10^{4}$ iterations. The comparison along rows shows the effect of increasing the sequence length, *L*, from 10 kb (**A**,**C**) to 100 kb (**B**,**D**). The comparison along the columns shows the effect of increasing the rate of recombination per nucleotide, *ρ*, from 0 (**A**,**B**) to $2\times 10^{-4}$ (**C**,**D**). See Section 2.4 for details.

To quantify the correlation between classical bootstrap and the two alternative support values, pairwise bootstrap and quartet analysis, we simulated $10^{4}$ samples of 20 related sequences 100 kb or 1 Mb long. To perturb sequence evolution, and thus increase the frequency of clades with low bootstrap support, we also added recombination. Figure 7 shows the average values of pairwise bootstrap and quartet analysis as a function of classical bootstrap values. In all cases, the agreement between pairwise bootstrap is closer to the ideal diagonal, and hence, classical bootstrap, than quartet analysis. This agreement improves as recombination is added; compare, for example, Figure 7A to Figure 7C. The difference in fidelity between pairwise bootstrap and quartet analysis becomes particularly conspicuous for the long sequences in Figure 7D, where the correlation between the raw classical and pairwise bootstrap values is 0.967, while that between classical bootstrap and quartet analysis is only 0.562.

An important aspect of our simulations not represented in Figure 7 is that the proportion of clades with a particular support value is not uniform across all possible outcomes. For example, in Figure 7D, 65.8% of quartet support values and 46.6% of pairwise bootstrap support values are maximal (not shown).

### 3.3. Application to Real Sequence Data

#### Human Mitochondrial Genomes

**Figure 8:**
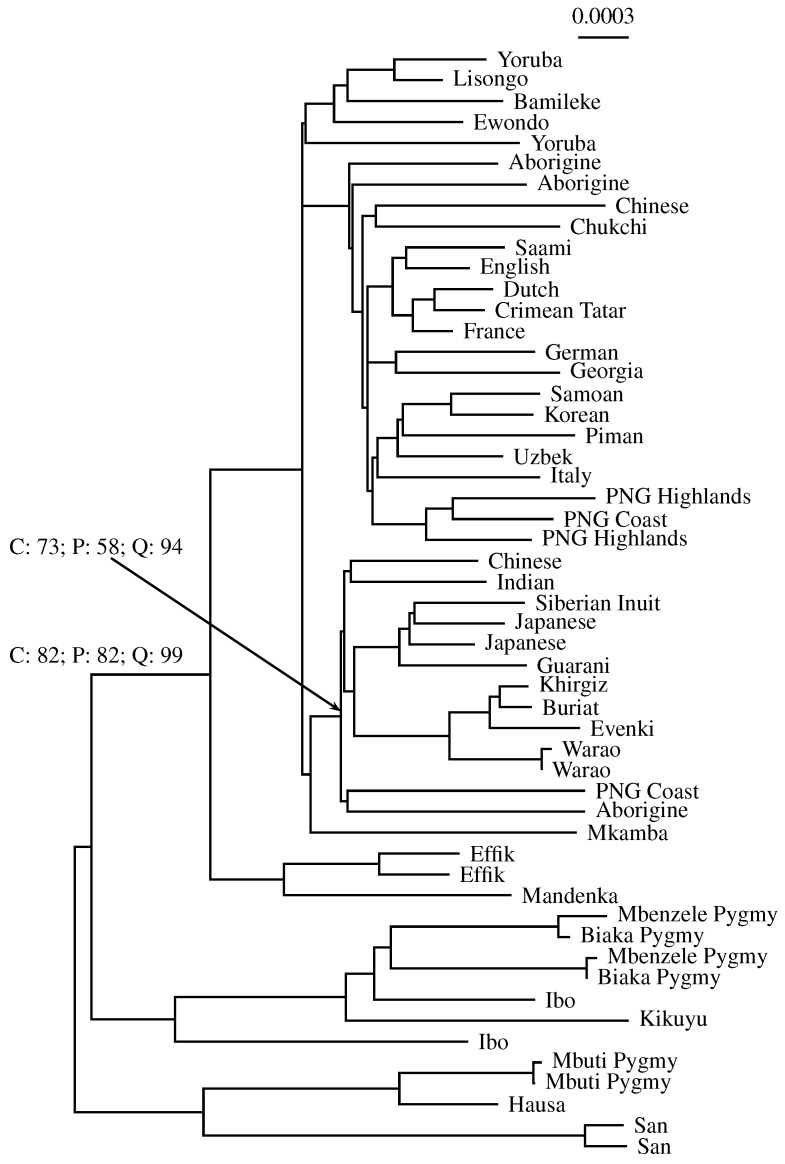
Phylogeny of humans computed from 53 complete mitochondrial genomes [14]. Example bootstrap support values are quoted for two nodes: C: classical alignment-based; P: pairwise bootstrap of andi distances; Q: quartet analysis of andi distances.

We investigated a sample of 53 complete human mitochondrial genomes originally collected to help resolve the geographic origin of humans [14]. Figure 8 shows the midpoint-rooted neighbor-joining tree based on these sequences. For two example nodes, we quote the percent support values for classical bootstrap (C), pairwise bootstrap (P) and quartet analysis (Q). The node pointed to by the arrow has a classical bootstrap value of 73%. This is reflected by the pairwise bootstrap support of 58%, but not by the close to perfect quartet support of 94%. Similarly, the other annotated node has a classical bootstrap value of 82%, which is identical to the pairwise bootstrap support. Again, quartet analysis gives over-optimistic support (99%).

**Figure 9:**
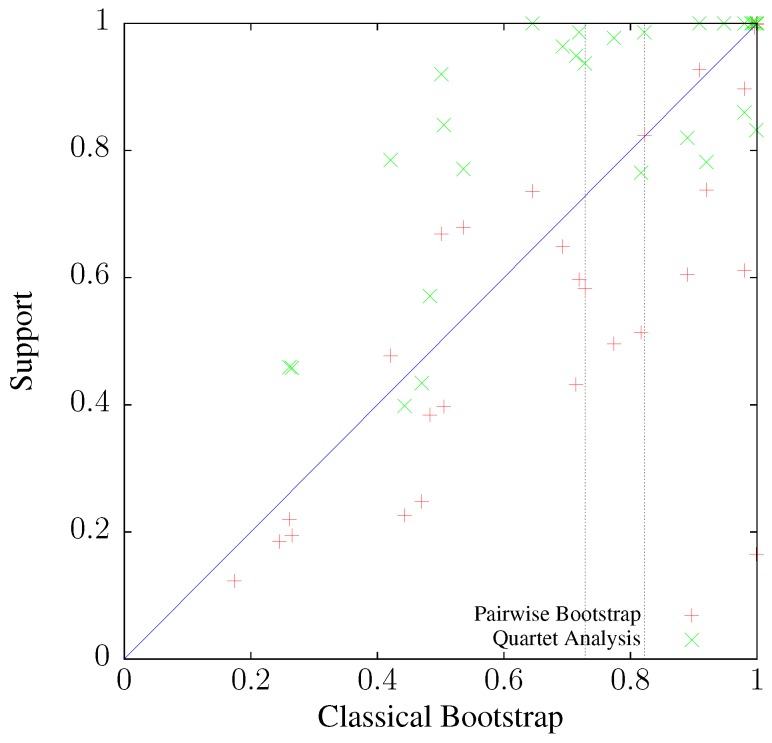
Correlation between bootstrap support values computed by classical bootstrap, pairwise bootstrap, and quartet analysis for the tree of 53 human mitochondrial genomes (Figure 8). Vertical lines: example nodes marked in Figure 8.

To investigate the relationship between classical bootstrap and its two alternatives further, the alternatives are plotted as a function of classical bootstrap in Figure 9. The vertical lines mark the two nodes annotated in Figure 8. Like in the simulations, it appears that pairwise bootstrap is more similar to its classical version than quartet analysis. Indeed, the correlation between classical and pairwise bootstrap is 0.829 ($P=4\times 10^{-11}$), while that between classical bootstrap and quartet analysis is only 0.777 ($P=6\times 10^{-9}$). Notice also the clustering of points in the top right hand corner of the graph, corresponding to a high frequency of clades with maximal support. This phenomenon was already noted when discussing the simulations shown in Figure 7.

**Figure 10:**
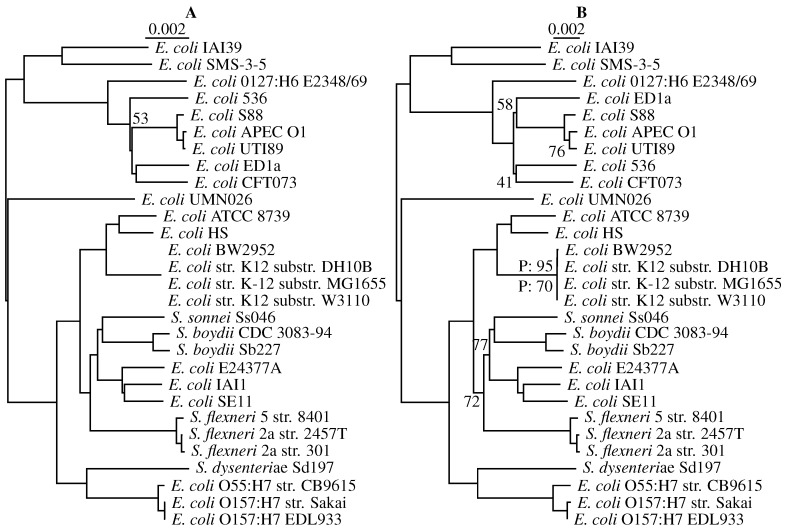
Phylogeny of 29 strains of *Escherichia coli*/*Shigella* computed from their full genomes. (**A**) Alignment-based; (**B**) andi-distances; the numbers refer to bootstrap support less than 100%; P: pairwise bootstrap; unmarked values in (B) refer to quartet support.

The program andi is designed for analyzing closely-related genomes, which are increasingly often collected in the course of pathogen outbreaks. For our second and final empirical example, we therefore use a benchmark set of 29 *E. coli*/*Shigella* genomes. Figure 10A shows the tree computed from alignment-based distances. Bootstrapping this alignment yields only a single clade with support less than 100%. This clade has a bootstrap support of 53 and comprises six uropathogenic *E. coli* strains thought to be affected by horizontal gene transfer [17]. Interestingly, the uropathogenic clade also contains the only major topological difference to the tree computed from andi-distances in Figure 10B: strains 536 and ED1a have switched positions. However, pairwise bootstrap fails to flag this clade; the only clades with pairwise bootstrap values less than 100% are part of the cluster of four very similar K12 strains. Quartet analysis, on the other hand, returns non-maximal support values even outside the K12 clade. In particular, it flags the group of uropathogenic strains. Note here that classical bootstrap evaluates individual nodes, while quartets refer to edges. In the case of the uropathogenic bacteria, the two outgoing edges are flagged by quartet analysis, as desired. In addition, quartet analysis indicates that two more clades in the *flexneri*/*sonnei* clade might be problematic with support values of 72% and 77%.

The inability of pairwise bootstrap to flag the uropathogenic clade is probably due to the fact that the estimation error of andi is large compared to the variance of the number of mismatches per site when bootstrapped from megabase-long genomes, such as those of *E. coli*. One indicator of the error in andi measurements is the difference between the estimates based on the two possible query/subject labellings [8]. For *E. coli*, this is often a few percent (not shown). Compare this to a mismatch rate of, say, 1% between two typical *E. coli* genomes of length 5 Mb. The variance of the number of mismatches is $5\times 10^{6}\times 0.01\times 0.99\approx 5\times 10^{4}$, and the standard deviation of the per site mismatch rate is $\sqrt{10^{4}}/\left(5\times 10^{6}\right)=2\times 10^{-5}$. In other words, 95% of the bootstrapped mismatch rates fall within an interval of $0.01\pm 4\times 10^{-5}$. Note that the numerator of the standard deviation is proportional to the square root of the sequence length, while the denominator is proportional to the untransformed sequence length. As a result, bootstrap variation decreases with sequence length.

## 4. Conclusions

Our new pairwise bootstrap scheme for andi emulates classical bootstrap values. With simulated data, the fit is quite good. With real data, where the estimation error is greater, classical bootstrap values are still approximated for short sequences, such as human mitochondrial genomes, which comprise approximate 16.6 kb. However, for longer sequences, such as *E. coli* genomes (5 Mb), the error in estimating evolutionary distances using andi can overwhelm the sensitivity of the pairwise bootstrap. In this situation, quartet analysis may be a suitable alternative. A topological difference between data and tree remains detectable by quartet analysis regardless of the size of the dataset, making this method immune to the saturation of the bootstrap with large samples. Our implementation of quartet analysis, afra, is efficient enough to analyze distance matrices for thousands of taxa in a few hours.
